# Synergistic Integration of Maize Biochar and *Bacillus amyloliquefaciens* Modulates Rhizosphere Bacterial Communities and Enhances Tomato Yield

**DOI:** 10.3390/microorganisms14050979

**Published:** 2026-04-27

**Authors:** Lin Wang, Yuanfeng Tian, Jiandong Jiang, Cansheng Yuan, Yingchun Du, Yuqi Song

**Affiliations:** 1 College of Rural Revitalization, Jiangsu Open University, Nanjing 210036, China; wanglin@jsou.edu.cn (L.W.); yuancs@jsou.edu.cn (C.Y.); duyc@jsou.edu.cn (Y.D.); 2 College of Life Sciences, Nanjing Agricultural University, Nanjing 210095, China; 15610055661@163.com (Y.T.); jiang_jjd@njau.edu.cn (J.J.)

**Keywords:** plant growth-promoting rhizobacteria, biochar, rhizosphere microbiota, synergistic mechanism, soil fertility

## Abstract

Integrating biochar with plant growth-promoting rhizobacteria (PGPR) is a promising strategy for sustainable soil management; however, the synergistic mechanisms governing rhizosphere microbial assembly remain inadequately understood. In this study, we investigated the combined effects of maize biochar (YM) and *Bacillus amyloliquefaciens* (BA) on tomato performance, soil physicochemical properties, and bacterial community dynamics via a controlled pot experiment. The results demonstrated that the synergistic treatment (YMBA) significantly enhanced tomato yield by 18.3% compared to the control, outperforming individual applications. This promotion was coupled with a comprehensive improvement in soil fertility, characterized by significant increases in soil organic matter (SOM), available nutrients (N, P, and K), and the activities of urease and acid phosphatase. High-throughput sequencing revealed that YMBA treatment significantly restructured the rhizosphere bacterial community, significantly increasing microbial richness and diversity. Notably, the synergistic application promoted the recruitment of beneficial taxa, particularly within the phylum Pseudomonadota. Mantel test analysis further elucidated that SOM and available phosphorus (AP) were the primary environmental drivers shaping the bacterial community turnover. Our findings suggest that biochar acts as a functional niche that facilitates *B. amyloliquefaciens* colonization and modulates the indigenous microbiota, providing a theoretical framework for utilizing cross-trophic synergies to optimize crop productivity and soil health.

## 1. Introduction

Plant Growth-Promoting Rhizobacteria (PGPR) have great potential in sustainable agriculture, as they enhance plant growth directly through mechanisms such as biological nitrogen fixation, phosphorus and potassium solubilization, and phytohormone production (e.g., auxins, cytokinins), and indirectly by suppressing pathogens and inducing systemic resistance [1,2]. However, the colonization and effectiveness of PGPR are often limited by low soil fertility or intense competition from indigenous microbial communities [3,4].

Biochar, a carbon-rich solid produced by pyrolyzing biomass under limited or no oxygen, offers a promising means to overcome these limitations [5,6]. It contains abundant inorganic nutrients and is widely applied to soil as an amendment. Importantly, the fine porous structure of the original biomass is well retained during pyrolysis, and the presence of functional groups such as hydroxyl, carboxyl, and aromatic rings endows biochar with a large specific surface area, strong adsorption capacity, and notable antioxidant activity [7,8]. These characteristics support its broad application in agriculture, industry, and environmental remediation.

In agricultural systems, biochar improves soil aggregate structure, enhances water and nutrient retention, modulates microbial abundance and community composition—often promoting beneficial microorganisms—and supplies nutrients for crop growth. Moreover, biochar can enhance the stability and persistence of PGPR through biosorption and biomineralization processes [9,10]. Unlike chemical stabilizers, biochar ameliorates soil properties without introducing contaminants. Thus, combining biochar with PGPR may simultaneously alleviate nutrient limitations and microbial competition while improving the colonization and functionality of beneficial microorganisms. Despite these potential advantages, the mechanistic links between soil environmental changes induced by biochar–PGPR coapplication and the resulting assembly of the rhizosphere microbiota remain poorly understood.

In this study, we hypothesized that biochar–PGPR coapplication promotes tomato growth by synchronously enhancing soil fertility and stimulating key nutrient-cycling enzyme activities, which collectively facilitates the recruitment of beneficial microbial taxa to optimize the rhizosphere bacterial community structure. To further investigate this synergy, a greenhouse experiment was conducted to examine the effects of biochar and PGPR (*Bacillus amyloliquefaciens*) on plant performance, soil physicochemical properties, and bacterial community. The specific aims were to: (i) explore the influences of biochar–PGPR coapplication on plant performance; (ii) elucidate the impact of coapplication on soil physicochemical properties; and (iii) evaluate the effects of coapplication on soil bacterial diversity and composition.

## 2. Materials and Methods

### 2.1. Characterization of Maize Biochar

The biochar applied in this study was synthesized from maize stalks, sourced from Zhengzhou Dingyi Environmental Technology Co., Ltd. (Zhengzhou, China). The material possessed the following core physicochemical characteristics: a Brunauer–Emmett–Teller (BET) surface area of 13 m^2^ g^−1^, and an average pore diameter of 15.8 nm. The carbonized product contained 25.67 g kg^−1^ sodium, 1.45 g kg^−1^ phosphorus, 41.17 g kg^−1^ potassium, 508.18 g kg^−1^ silicon and 110.63 g kg^−1^ iron.

### 2.2. Greenhouse Experiment

The greenhouse experiment was conducted at Nanjing Agricultural Science Research Institute in Jiangsu Hilly Region. Natural soil used for the experiment was collected from Xianlin Street, Nanjing, China (118°92′ E, 32°09′ N); the soil was classified as yellow-brown soil. Tomato seeds (Red Dwarf variety [11]) underwent surface sterilization, germination, and seedling cultivation before transplantation. *Bacillus amyloliquefaciens* was cultured in Luria–Bertani (LB) broth at 30 °C with shaking at 180 rpm for 24 h. After cultivation, the bacterial cells were harvested by centrifugation (5000× *g*, 10 min), washed twice with sterile saline (0.85% NaCl), and resuspended in sterile distilled water. The cell density was adjusted to 1.0 × 10^6^ CFU/mL based on optical density (OD_600_) measurements and confirmed by serial dilution plating. The treatments were as follows: (1) CK (untreated); (2) YM: 5 g of biochar was thoroughly mixed with 500 g of soil before potting; (3) BA (which shows the ability to fix nitrogen, solubilize phosphate and potassium, and produce IAA [12]): a liquid inoculum containing 1.0 × 10^6^ CFU/mL was mixed uniformly with 500 g of soil and then filled into pots. (4) YMBA: the liquid inoculum (1.0 × 10^6^ CFU/mL) was first blended with 5 g of biochar, and the resulting mixture was then incorporated into 500 g of soil and homogenized before potting. Each treatment had four replicates (one plant per replicate).

### 2.3. Determination of Plant Performance

The yield was assessed over a 30-day period, commencing at 60 days after tomato transplantation. Then, the plants were harvested to determine the shoot/root length. The shoot/root dry weight of all plant samples were first dried at 105 °C for 30 min and then at 70 °C for 5 days.

### 2.4. Soil Physicochemical Properties Determination

Soil pH and EC was measured using a glass electrode meter with a soil-to-water ratio of 1:5 (*w*/*v*). Soil organic matter (SOM) was determined using the potassium dichromate external heating method. Available potassium (AK), available phosphorus (AP) and alkali-hydrolyzable nitrogen (AN) were measured following previous protocols [13].

### 2.5. Soil Enzyme Activity Determination

The activity of soil enzymes, including soil urease (SUE), soil sucrase (SSC) and soil acid phosphatase (SACP), were measured using the assay kit (Nanjing JianCheng Bioengineering Institute Co., Ltd., Nanjing, China) according to the provided instructions.

### 2.6. DNA Extraction, Bioinformatic and Statistical Analyses

DNA from each rhizosphere soil sample (0.3 g) was extracted using the PowerSoil DNA extraction kit (Mo Bio, Carlsbad, CA, USA) according to the provided instructions. The quality (A260/A280) and concentration of the extracted DNA were determined using NanoDrop (ThermoScientific, Wilmington, DE, USA). The DNA samples were then sent to Shanghai Ling En Biotechnology Co., Ltd. (Shanghai, China) for sequencing.

The V4 region of the 16S rRNA gene was PCR-amplified to investigate bacterial communities using the primer set 563F (5′-AYTGGGYDTAAAGVG-3′) and 802R (5′-TACNVGGGTATCTAATCC-3′). 16S rRNA gene sequence data was processed with the UPARSE pipeline as described previously with the following criteria: (i) The 250 bp reads were truncated at any site receiving an average quality score < 20 over a 10 bp sliding window, discarding the truncated reads that were shorter than 50 bp. (ii) Exact barcode matching, 2 nucleotide mismatch in primer matching, and reads containing ambiguous characters were removed. (iii) Only sequences that overlapped longer than 10 bp were assembled according to their overlap sequence. Reads that could not be assembled were discarded. Passed sequences were dereplicated and subjected to the DADA2 algorithm (QIIME 2) to identify indel-mutations and substitutions. The trimming and filtering were performed on paired reads with a maximum of two expected errors per read (maxEE  =  2). After merging paired reads and chimera filtering, the phylogenetic affiliation of each 16S rRNA gene sequence (herein called ASVs) was analyzed by RDP Classifier (https://github.com/rdpstaff/classifier?utm_source, accessed on 19 May 2025) against the Silva (SSU132)16S rRNA database using a confidence threshold of 70%.

Diversity analysis on bacterial communities was performed using the vegan package [14] in R (v 4.5.1). A principal coordinate analysis (PCoA) based on weighted Unifrac distances was performed to explore the differences in bacterial community structures among different treatments. For the separate in-depth analyses of each sample type, we additionally applied the following sequence count threshold to the ASVs tables: we selected ASVs with at least two sequences (avoiding single-count ASVs) that were present in at least four samples (the number of replicates per treatment). Linear Discriminant Analysis Effect Size (LEfSe) was performed using the microeco package in R to identify differentially abundant microbial taxa among different treatments.

Experimental data processing utilized statistical analysis software including SigmaPlot (v 14) and R (v 4.5.1). Analysis of variance (ANOVA) was used to compare mean differences among the treatments. A principal coordinate analysis (PCoA) based on weighted Unifrac distances was performed in R (v 4.5.1) to explore the differences in bacterial community structures among the treatments.

## 3. Results

### 3.1. The Influences of Different Treatments on Plant Performance

**Figure 1:**
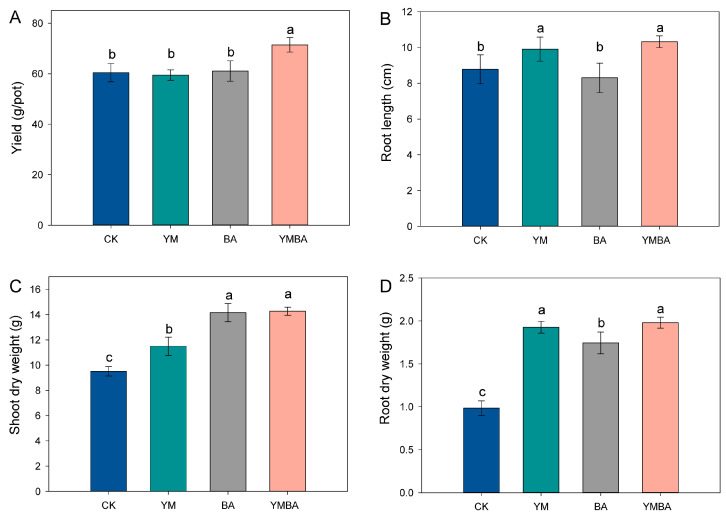
Effect of different treatments on (**A**) yield, (**B**) root length, (**C**) shoot and (**D**) root dry weight. CK: Control treatment, YM: maize biochar, BA: *B. amyloliquefaciens*, YMBA: maize biochar and *B. amyloliquefaciens*. Means followed by different letters are statistically different from each other according to Duncan’s Multiple Range test at *p* < 0.05.

The application of either biochar or PGPR significantly enhanced plant growth during the tomato growth period (Figure S1). Compared to the CK treatment, biochar amendment significantly increased shoot length, shoot weight, and root weight. In contrast, *B. amyloliquefaciens* inoculation specifically enhanced root length (Figure 1). The YMBA treatment significantly increased shoot and root length and shoot and root biomass by 18.1%, 17.5%, 50.1%, 101%, respectively. YMBA treatment not only promoted plant growth but also increased tomato yield by approximately 18.3%.

### 3.2. The Influences of Different Treatments on Soil Physicochemical Properties

YMBA significantly enhanced the contents of several soil nutrients (Table 1). Compared with the CK treatment, the YM treatment significantly increased soil organic matter (SOM) content by 107.6%, but did not significantly alter other parameters. The BA treatment significantly elevated electrical conductivity (EC) by 27.7%, available phosphorus (AP) by 14.8%, available potassium (AK) by 27.2%, and alkaline-hydrolyzable nitrogen (AN) by 22.2% relative to CK treatment. Notably, the YMBA treatment demonstrated comprehensive improvement: it significantly increased EC by 78.4%, SOM by 111.5%, AP by 26.4%, AK by 28.7%, and AN by 21.8% compared to CK. No significant differences in soil pH were observed among all treatments.

### 3.3. The Influences of Different Treatments on Soil Enzyme Activity

The activities of urease, acid phosphatase, and sucrase are associated with key soil nutrients, including carbon, nitrogen, and phosphorus (Table 2). Biochar amendment significantly increased the activities of soil acid phosphatase and urease compared with CK treatment. The application of biochar and *Bacillus amyloliquefaciens* did not significantly affect soil sucrase activity. Compared with CK treatment, YMBA treatment showed a stronger and more comprehensive stimulatory effect, significantly increasing soil urease (SUE) activity by 31.5% and soil acid phosphatase (SACP) activity by 25.2%. YM treatment specifically enhanced SACP activity by 15.1%, while the BA treatment did not significantly alter the activities of any measured enzymes. No significant differences in soil sucrase (SSC) activity were observed among all treatments.

### 3.4. The Influences of Different Treatments on Bacterial Communities

**Figure 2:**
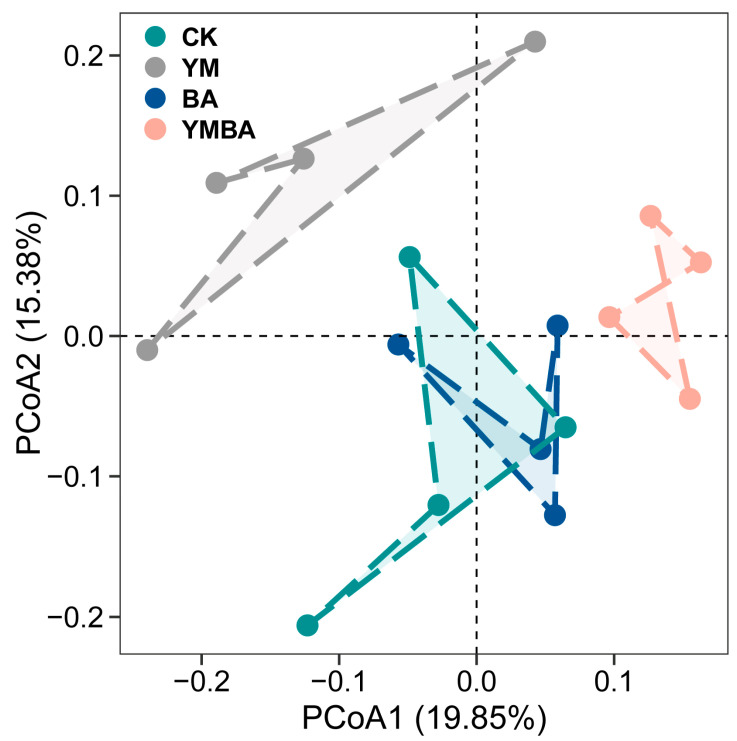
Effects of different treatments on microbial communities. Principal coordinate analysis (PCoA) of bacterial community structure. CK: Control treatment, YM: maize biochar, BA: *B. amyloliquefaciens*, YMBA: maize biochar and *B. amyloliquefaciens*.

**Figure 3:**
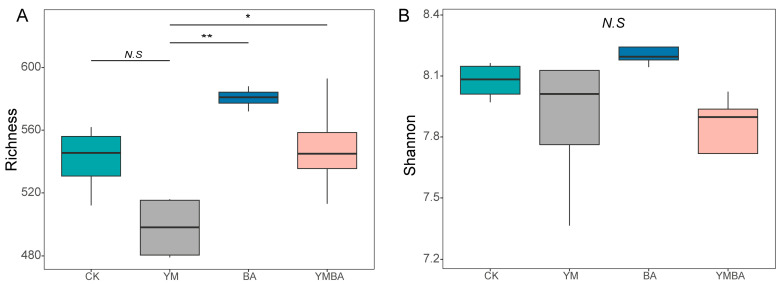
Effects of different treatments on (**A**) microbial richness and (**B**) diversity. CK: Control treatment, YM: maize biochar, BA: *B. amyloliquefaciens*, YMBA: maize biochar and *B. amyloliquefaciens*.* indicates *p* < 0.05, ** indicates *p* < 0.01. *N.S* indicates not significant.

**Figure 4:**
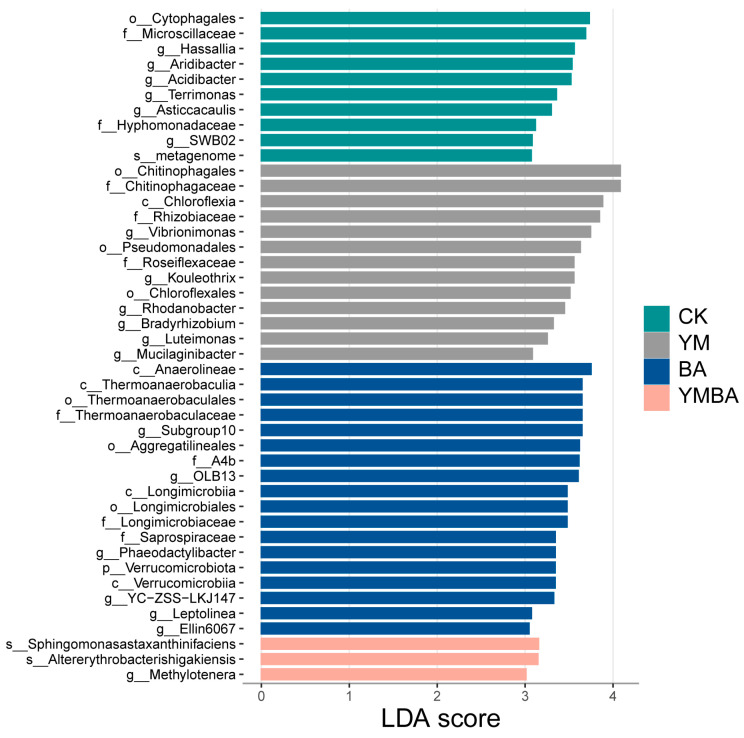
Least discriminant analysis of microbe of different treatments. CK: Control treatment, YM: maize biochar, BA: *B. amyloliquefaciens*, YMBA: maize biochar and *B. amyloliquefaciens*.

The addition of biochar led to significant alterations in the community structure of the rhizosphere bacteria (ANOSIM test, *p* < 0.001, Figure 2). As for α-Diversity, we found that the addition of biochar led to higher bacterial richness but no significant changes for the bacterial Shannon index (*p* > 0.05, Figure 3). We selected the top 9 most abundant bacterial phyla (Acidobacteriota, Actinomycetota, Bacillota, Bacteroidota, Chloroflexota, Cyanobacteriota, Gemmatimonadota, Patescibacteria and Pseudomonadota) while the remaining phyla were grouped into “Others”, and found that Pseudomonadota was enriched by biochar and *B. amyloliquefaciens* compared to the CK, YM or BA treatments but not significant among the treatments (Figure S2). The least discriminant analysis (LDA) showed differential species of tomato across the four treatments. The CK, YM, BA and YMBA treatments had 10, 13, 18 and 3 biomarkers, respectively (Figure 4).

### 3.5. The Linkages Between Bacterial Abundance/Plant Performance and Soil Nutrients

**Figure 5:**
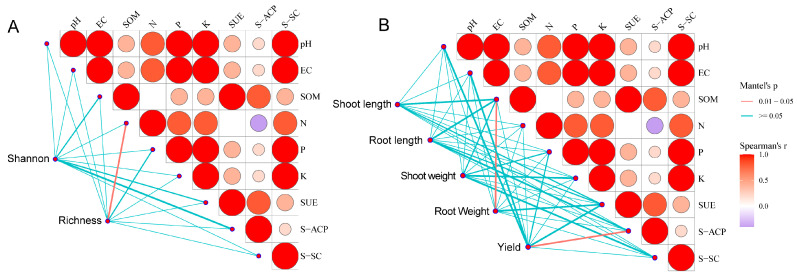
Relationship between microbial α-diversity (**A**) or plant growth indexes (**B**) and soil physicochemical properties based on Mantel test analysis. Edge width corresponds to the Mantel r statistic of the corresponding distance correlation.

We analyzed microbial abundance, diversity, and soil physicochemical properties and found that microbial richness was positively correlated with soil organic matter and alkaline-hydrolyzable nitrogen. Furthermore, pH exhibited significant negative correlations with electrical conductivity (EC) and available potassium (AK), while demonstrating a positive association with soil organic matter (SOM). Enzyme activities, including soil urease (SUE), soil acid phosphatase (S-ACP), and soil sucrase (S-SC), were generally positively correlated with soil nutrient contents such as available phosphorus (AP) and AK. Furthermore, both the Shannon and Richness diversity indices displayed moderate to strong positive relationships with key soil fertility indicators like SOM and P, as well as with the measured enzyme activities (Figure 5A).

Soil provides the essential environment for plant growth. Therefore, we analyzed the relationships between plant performance and soil physicochemical properties. The results revealed that root dry weight exhibited positive correlations with both EC and soil organic matter. Meanwhile, the activities of soil urease and acid phosphatase were positively correlated with yield (Figure 5B).

## 4. Discussion

### 4.1. Combination of Biochar and PGPR Can Promote Plant Performance

The biochar–PGPR coapplication significantly enhanced plant growth. The biochar–PGPR coapplication increased tomato yield and soil nutrient content. The combined application of biochar-based Paraburkholderia phytofirmans and *Bacillus* sp. significantly increased grain yield (14%) [15]. The study showed that a microbial consortium immobilized in biochar increased the soybean yield by 18.75% compared to the CK group. The yield was indirectly promoted by improving root nutrient uptake efficiency [16].

Our study showed that biochar–PGPR coapplication increased shoot, root length and shoot, root biomass, and biochar–PGPR coapplication resulted in a more substantial increase in tomato yield relative to the individual amendment of either biochar or PGPR alone (Figure 1).

Under the experimental conditions of this study, the individual application of PGPR or biochar did not significantly enhance tomato yield, which may be attributed to factors such as plant genotype variation, competition from indigenous microbiota, and the influence of specific edaphic factors [17]. These elements collectively contribute to the inconsistent or limited efficacy of biochar and PGPR amendments.

### 4.2. Effects of Different Treatments on Soil Physicochemical Properties and Soil Enzyme Activity

Soil physicochemical properties are closely associated with plant performance. In our study, compared with the CK treatment, the biochar–PGPR coapplication significantly increased soil electrical conductivity, the concentrations of alkali-hydrolyzable nitrogen, available phosphorus, and available potassium, as well as soil organic matter and the activities of soil acid phosphatase and urease. These results are consistent with previous findings [18]. While potential trade-offs like nutrient immobilization can occur at higher dosages, our results suggest that a 1% application rate remains within a beneficial threshold for this system [19].

In addition, YMBA treatment demonstrated synergistic benefits compared to the individual YM and BA treatments by combining their respective advantages into a more effective soil amendment strategy. Relative to YM, YMBA maintained similarly high levels of soil organic matter while significantly increasing the availability of key nutrients, including alkali-hydrolyzable nitrogen, available phosphorus, and available potassium, which may be attributed to the ability of *B. amyloliquefaciens* to perform nitrogen fixation, phosphorus solubilization, and potassium mobilization [20]. Compared to BA, YMBA substantially enhanced EC and improved soil organic matter content, while retaining the nutrient solubilizing capacity contributed by the PGPR. Thus, the combined treatment integrated the nutrient retention and substrate improvement of biochar with the nutrient mobilizing function of *B. amyloliquefaciens*. This created a more balanced and fertile soil environment. Consequently, it supported plant growth better than either amendment alone. Urease and phosphatase activities correlate with soil nitrogen and phosphorus hydrolysis, respectively. Different biochar amendments enhanced soil enzyme activities by serving as a nutrient-rich carrier that improved the environment, thus promoting microbial and plant growth and metabolism [21,22]. The combined application of biochar and PGPR significantly increased soil nitrate-nitrogen content and resulted in a 15.97% higher average concentration throughout the entire growth period compared to the biochar-alone treatment. Our study also found the combined application of biochar and PGPR increased soil alkali-hydrolyzable nitrogen content. Practical application of microbial inoculants (e.g., *Bacillus* sp. NL-11) has demonstrated their ability to significantly increase plant biomass by enhancing soil organic carbon, available phosphorus, and the activities of multiple enzymes (e.g., urease, phosphatase, β-glucosidase) [23].

### 4.3. Differences in Microbial Communities Among the Treatments

The structure and composition of soil microbial communities represent the functional core of terrestrial ecosystems, where shifts in their diversity and stability directly influence plant nutrient acquisition and health status [24]. According to α- and β-diversity results, the addition of biochar led to significant alterations in the community structure of the rhizosphere bacteria (Figure 2). Tight feedback mechanisms exist between soil microorganisms and plants. Plants regulate the composition and function of rhizosphere microbial communities through root exudates, while microorganisms promote plant growth via multiple strategies, including the production of phytohormones, nitrogen fixation, phosphorus solubilization, and suppression of pathogens. Conversely, the composition and structure of the rhizosphere microbial community are crucial for plant performance. Significant different soil microbiome composition associated with healthy and diseased plants was observed during plant growth [25,26].

Our results showed biochar–PGPR coapplication increased potential plant-beneficial microbiomes such as Pseudomonadota. Various bacteria within the phylum Pseudomonadota can promote plant growth by facilitating biological nitrogen fixation. They directly suppress soil-borne pathogens through the production of antibiotics and secretion of cell wall-degrading enzymes, while simultaneously priming plant immune defenses for enhanced responsiveness to subsequent pathogen challenges.

The increases in SOM and AP induced by biochar provided abundant substrates for copiotrophic microorganisms, enabling them to gain a competitive advantage within the rhizosphere niche [27]. These taxonomic shifts exerted a direct feedback on soil function, as evidenced by the significant elevation of soil phosphatase and urease activities. Through the interlocking reactions among environmental factors, microbial communities, and enzymatic metabolism, the biotransformation of soil mineral elements was significantly promoted, thereby establishing a nutrient-rich microenvironment conducive to plant development [28].

### 4.4. Linkages Between Soil Environmental Properties and α-Diversity or Plant Performance

Soil physicochemical properties establish the foundation for microbial community structure and function. The factors such as soil pH, organic matter, and nutrient availability significantly influence microbial composition and abundance. For instance, long-term nitrogen fertilizer application alters soil microbial community structure, reducing diazotroph populations while increasing nitrifying and denitrifying bacteria [29]. Furthermore, soil enzyme activities, serving as key indicators of microbial metabolic activity, are regulated by both soil physicochemical properties and the microbial community.

We found that acid phosphatase and urease activities correlated positively with crop yield, underscoring the importance of microbially driven nutrient cycling in plant productivity [30]. In nutrient cycling, the activities of acid phosphatase and β-glucosidase are closely associated with phosphorus and carbon transformations, respectively, and these enzymatic activities are driven by soil organic matter and nutrient status [31]. Microbial communities further influence nutrient mineralization and supply by modulating soil enzyme activities, thereby affecting plant growth. Microbially produced hydrolases convert organic nutrients into plant-available inorganic forms. For example, plants stimulate microbial activity through root exudates, consequently enhancing enzyme activities and promoting the mobilization of nutrients such as nitrogen and phosphorus [32] in the rhizosphere.

## 5. Conclusions

In summary, this study demonstrates that the combined application of maize biochar and *Bacillus amyloliquefaciens* offers a superior strategy for enhancing tomato productivity and soil health compared to individual amendments. The synergistic effect is primarily driven by dual mechanisms: (i) the improvement of soil physicochemical properties, specifically, the increase in SOM and available nutrients (N, P, K); and (ii) the modulation of the rhizosphere bacterial community, characterized by the enrichment of beneficial Pseudomonadota and enhanced activities of soil urease and acid phosphatase. Our findings suggest that biochar serves as an effective carrier that facilitates the colonization and function of PGPR. Consequently, the “Biochar + PGPR” integrated management strategy represents a promising, sustainable approach to alleviating soil degradation and improving crop yields in agricultural production. Future research should focus on: (1) validating these findings in long-term field trials across different agroecological zones and soil types to assess stability and scalability; (2) applying multi-omics approaches (e.g., metagenomics, metatranscriptomics, and metabolomics) to decipher the molecular crosstalk between biochar-derived signals and PGPR gene expression; and (3) evaluating the performance of this strategy under abiotic stress conditions such as drought, salinity, or nutrient deficiency.

## Figures and Tables

**Table 1:** Effect of different treatments on soil physicochemical properties.

Treatment	EC (us cm^−1^)	pH	SOM (g kg^−1^)	AP (mg kg^−1^)	AK (mg kg^−1^)	AN (mg kg^−1^)
CK	398 ± 19.22 c	6.83 ± 0.05 b	21.04 ± 5.68 c	9.23 ± 0.31 b	185.75 ± 5.68 b	218.31 ± 11.19 b
YM	390 ± 18.78 c	6.82 ± 0.03 b	43.67 ± 2.62 a	8.09 ± 0.34 b	184.25 ± 3.77 b	257.25 ± 10.88 a
BA	508.25 ± 21.17 b	6.87 ± 0.04 b	33 ± 5.59 b	10.60 ± 0.30 a	236.25 ± 3.30 a	266.88 ± 4.97 a
YMBA	710 ± 22.46 a	6.95 ± 0.07 ab	44.5 ± 2.57 a	11.67 ± 1.40 a	239 ± 5.35 a	266 ± 8.57 a

CK: Control treatment, YM: maize biochar, BA: *B. amyloliquefaciens*, YMBA: maize biochar and *B. amyloliquefaciens*. Means followed by different letters are statistically different from each other according to Duncan’s Multiple Range test at *p* < 0.05.

**Table 2:** Effect of different treatments on soil enzyme activity.

Treatment	SUE (mg g^−1^d^−1^)	SACP (mg g^−1^d^−1^)	SSC (mg g^−1^d^−1^)
CK	0.73 ± 0.03 c	4.69 ± 0.39 b	7.07 ± 0.80 a
YM	0.86 ± 0.09 ab	5.40 ± 0.21 a	6.97 ± 0.05 a
BA	0.81 ± 0.03 bc	4.23 ± 0.35 b	7.78 ± 0.68 a
YMBA	0.96 ± 0.07 a	5.87 ± 0.51 a	7.91 ± 0.49 a

CK: Control treatment, YM: maize biochar, BA: *B. amyloliquefaciens*, YMBA: maize biochar and *B. amyloliquefaciens*. Means followed by different letters are statistically different from each other according to Duncan’s Multiple Range test at *p* < 0.05.

## Data Availability

The original data presented in the study are openly available in NGDC at CRA038591.

## Supplementary Materials

The following supporting information can be downloaded at: https://www.mdpi.com/article/10.3390/microorganisms14050979/s1, Figure S1: Effect of different treatments on shoot length of at seedling stage, flowering stage and fruiting stage of tomato plant growth; Figure S2: Relative abundance of microbe at the phylum level of different treatments.
