# Supplementary figures and images for: Synergistic Integration of Maize Biochar and Bacillus amyloliquefaciens Modulates Rhizosphere Bacterial Communities and Enhances Tomato Yield
Source: Microorganisms. 2026 Apr 27;14(5):979. doi: 10.3390/microorganisms14050979 (PMC13209771; doi:10.3390/microorganisms14050979)

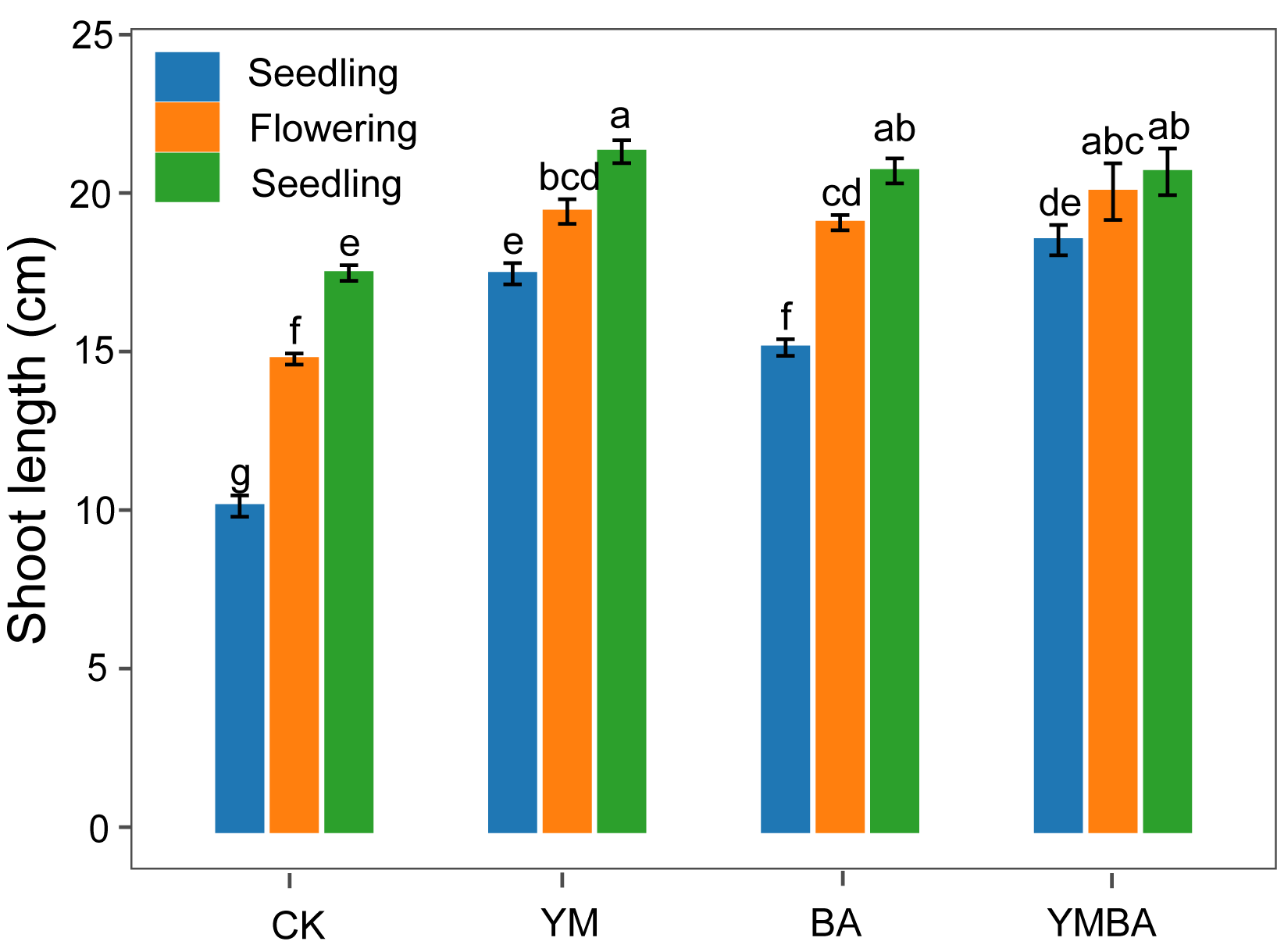

Supplement: Supplementary file 1 [file microorganisms-14-00979-s001.zip › Figure S1.tif]

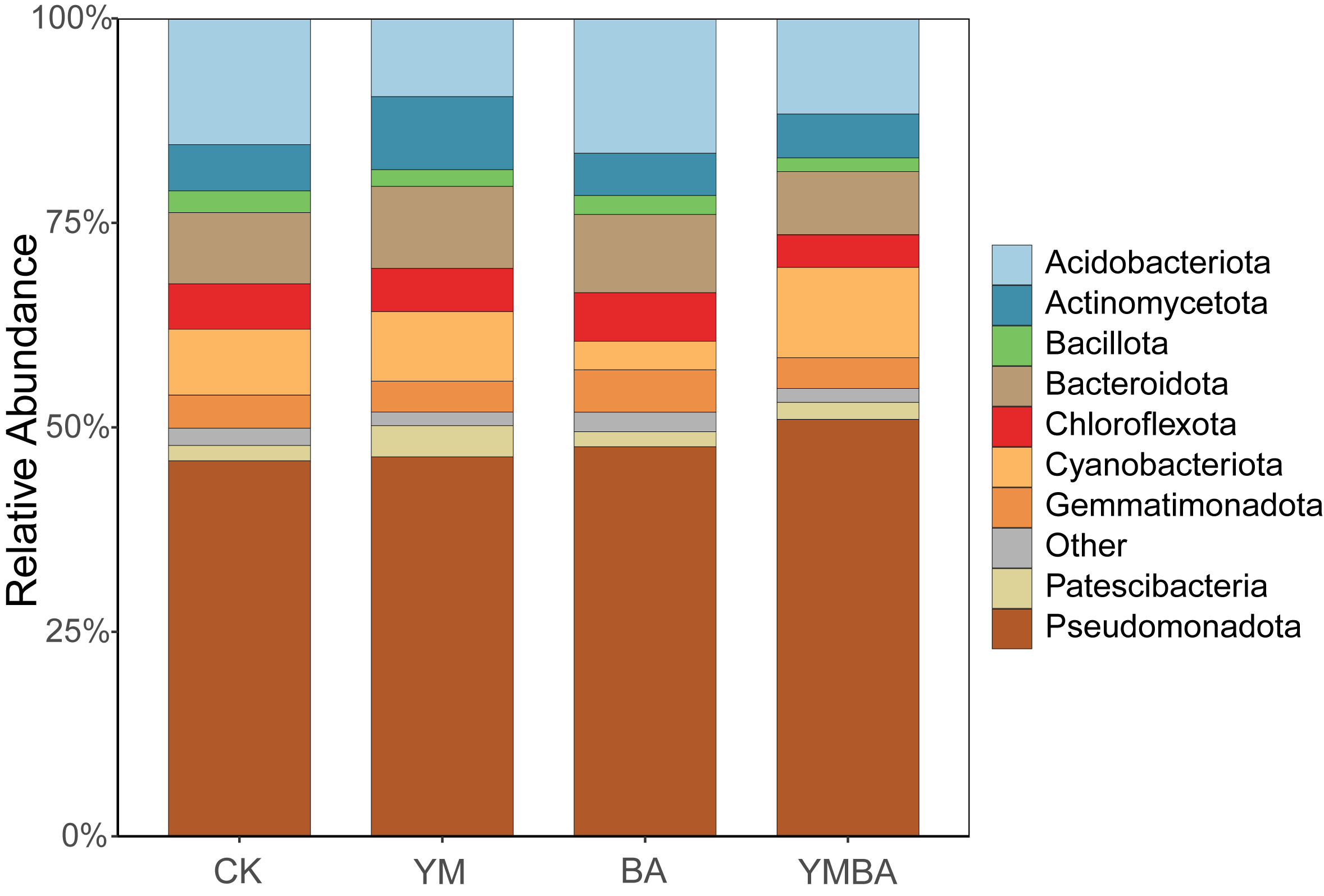

Supplement: Supplementary file 1 [file microorganisms-14-00979-s001.zip › Figure S2.tif]
